# Optimized cyclosporine starting dose may reduce risk of acute GvHD after allogeneic hematopoietic cell transplantation: a single-center cohort study

**DOI:** 10.1038/s41409-022-01598-6

**Published:** 2022-02-08

**Authors:** Jérémie Héritier, Michael Medinger, Dominik Heim, Helen Baldomero, Christian Arranto, Jörg P. Halter, Jakob R. Passweg, Martina Kleber

**Affiliations:** 1 University Hospital Basel, Division of Hematology, Basel, Switzerland; 2 University of Basel, Basel, Switzerland; 3 University Hospital Basel, Department of Internal Medicine, Basel, Switzerland

**Keywords:** Stem-cell research, Risk factors, Haematological cancer, Disease prevention

## Abstract

Cyclosporine A (CsA) is commonly used for Graft versus Host Disease (GvHD) prophylaxis at a recommended starting dose of 3 mg/kg/d: Evidence for the effect of different CsA starting doses on GvHD risk is limited. We therefore estimated the association of 5 mg/kg/d (CsA5) and 3 mg/kg/d (CsA3) CsA starting doses with GvHD risk in two consecutive cohorts of allogeneic hematopoietic cell transplantation (allo-HCT) patients, exploring potential risk factors for incident acute GvHD, with a focus on CsA starting dose. We analyzed 519 patients within CsA5 (*n* = 153) and CsA3 (*n* = 366). The cumulative incidence function of acute GvHD grade ≥2 was higher in the CsA3 compared to the CsA5 group (41% vs. 33%, respectively; *p* = 0.043), without impacting chronic GvHD. In multivariable analysis, a CsA starting dose of 3 mg/kg/d, no ATG use, unrelated donor and high to very high disease risk index were significantly associated with acute GvHD grade ≥2. A higher CsA starting dose of 5 mg/kg/d was independently associated with lower acute GvHD risk, and higher CsA levels in the early period after allo-HCT were reached.

## Introduction

Allogeneic hematopoietic cell transplantation (allo-HCT) offers a potential curative option for various hematological disorders [1–3], but is associated with significant morbidity and mortality [4, 5]. Acute (aGvHD) and chronic GvHD (cGvHD)–an alloreactivity against host antigens – is linked to morbidity and mortality after allo-HCT, limiting its beneficial effects [6–14]. Relevant risk factors for the development of GvHD are the patient age, donor type and HLA mismatch, stem cell source, donor-recipient sex constellation, disease activity, CMV serostatus, conditioning regimen and GvHD prophylaxis [15–19].

Cyclosporine (CsA), a calcineurin-inhibitor, is a commonly used drug for GvHD prophylaxis [20, 21]. Initial dosing, timing of CsA application, target CsA blood level concentrations and schedule of administration remains variable [22–34]. Optimal CsA dosing may be of importance as higher incidence of GvHD has been in previous studies associated with lower CsA exposure and that high CsA levels were associated with more graft failures in RIC transplants [14]. The recommended standard CsA starting dose by the EBMT handbook and used since the first clinical trials establishing the combination of CsA with methotrexate as standard GvHD prophylaxis is 3 mg/kg [34, 35]. Previously, we could demonstrate that close monitoring of CsA dosing with active adjustments to maintain therapeutic CsA levels >195 µg/L in the first 10 days after allo-HCT significantly may reduce the incidence of aGvHD [36]. It is unclear however, if higher CsA starting doses affect post-allo-HCT CsA levels and GvHD risk [22, 27, 36, 37].

Following the analysis of our previously published data, we changed starting dose from 3 mg/kg to 5 mg/kg over 6 h intravenously (i.v.) [36]. Here we report the potential impact of this measure on clinical outcomes.

## Material and methods

### Patient population and study design

This single-center cohort study included consecutive patients from the Department of Hematology of the University Hospital of Basel with (i) hematological diseases who had (ii) their first allo-HCT between January 2010 and October 2018. Patients with a CsA starting dose of 3 mg/kg/d from January 2010 to June 2016 were compared to patients who received consecutively a starting dose of 5 mg/kg/d CsA from June 2016 to October 2018.

We extracted relevant patient and disease characteristics from our electronic research database–including sex, age, HCT pretransplant risk scores (Karnofsky Performance Status [KPS], European Group for Blood and Marrow Transplantation [EMBT] score [38], Hematopoietic Cell Transplantation Comorbidity Index [HCT-CI] [39], disease risk index [DRI] [40]), and the interval diagnosis to allo-HCT. Allogeneic HCT characteristics include CMV status, donor type (HLA match/mismatch, related/unrelated), conditioning regimen, GvHD prophylaxis, stem cell source and CD34+ cell dose. Serum creatinine and estimated glomerular filtration rate (eGFR) by the CDK-EPI (Chronic Kidney Disease Epidemiology Collaboration) equation [41] were measured on day 0 (time of HCT) and on day 7 after HCT [42]. The study was approved by the Ethics Committee of Northwestern and Central Switzerland (EKNZ study number: 2018-02000). All patients provided their informed consent for further use of their data for research purposes.

The primary outcome of this study was incident aGvHD grade ≥2 following a first allo-HCT in patients with CsA starting dose of 3 mg/kg/d compared 5 mg/kg/d. Secondary outcome measures were 1-year progression-free (PFS) and -overall survival (OS), and the incidence of 1-year non-relapse-mortality (NRM) and -relapse.

### Graft-versus-host disease prophylaxis and conditioning regimens

Myeloablative conditioning (MAC) protocols consisted of cyclophosphamide combined with busulfan, cyclophosphamide and total body irradiation (TBI) ≥ 8 Gray, cytarabine, carmustine, etoposide, melphalan, and fludarabine (BEAM- fludarabine) and other protocols [36].

Reduced-intensity conditioning (RIC) protocols consisted of fludarabine with low-dose TBI < 6 Gy, fludarabin combined with busulfan or melphalan, and other protocols. Reasons for RIC were relevant comorbidities or advanced age [36].

Within MAC conditioning regimens, GvHD prophylaxis consisted of CsA and methotrexate (MTX) as well as anti-thymocyte globulin (ATG) in case of unrelated donors and in matched related donors ≥ 40 years [43] or other protocols: CsA was administered in a dose of 3 mg/kg/day and 5 mg/kg/day i.v. starting day-3 (in BEAM-fludarabin protocols on day -1, *n* = 20) before allo-HCT. Antit-hymocyte globulin (rabbit) was used in the dose of 5 mg/kg/d (day-3), 10 mg/kg/d (day -2) and 20 mg/kg/d (day -1) as previously described (Grafalon, Neovii ®) [43]. GvHD prophylaxis in patients with RIC conditioning regimens included CsA, MTX, and ATG in patients with unrelated donors and matched related donors ≥ 40 years (if RIC included fludarabin/busulfan), or CsA from day -1 and mycophenolat mofetil (MMF) if RIC was based on fludrarabin/low-dose TBI [36]. The measurement of CsA blood concentration was done as trough levels by high-performance liquid chromatography (HPLC) at least weekly, and in addition two days after dose changes. Target levels were 150-200 µg/L as per EBMT recommendations [35] with both CsA starting doses of 3 mg/kg/d and 5 mg/kg/d. During the post-allo-HCT period, CsA was changed from i.v. to oral (per os) given adequate oral intake and thereafter tapered and stopped 180 days after HCT in the absence of aGvHD.

### GvHD diagnosis and therapy

Acute GvHD was graded according to the modified Glucksberg criteria [44] and cGvHD was graded based on the revised Seattle criteria [45]. In case of clinical relevant GvHD grade ≥2, patients were treated with corticosteroids i.v. (methylprednisolone, 2 mg/kg/d). The definition of corticosteroid resistance was defined by no response after 7 days of treatment or clear progression after five days [46].

### Statistical analysis

Continuous variables were reported as median (interquartile range [IQR]). We assessed differences in demographic, clinical, and transplantation parameters using Chi-squared or Fisher’s exact test for categorical variables and Student’s *t*-test or Mann–Whitney *U* test for continuous variables, depending on data distributions. Categorical variables were presented as proportions. The cumulative incidence function of one-year NRM and relapse/progression were estimated by accounting for competing events. To study GvHD, death or relapse were considered as competing events. Probabilities of PFS and OS were estimated using the Kaplan–Meier estimator at one year.

We analyzed CsA starting doses of 3 mg/kg/d and 5 mg/kg/d in a multivariable model. Risk factors for aGvHD grade ≥2 were investigated by fitting univariable and multivariable subdistribution proportional hazards models by Fine&Gray; co-variables included the CsA starting doses of 3 mg/kg/d and 5 mg/kg/d, ATG vs. non-ATG based GvHD prophylaxis, donor/recipient gender, disease risk index, donor age, graft source, donor type and conditioning regimens [47]. In the final multivariable analysis, only significant confounders (at univariable *p* < 0.05) were included. For each patient, follow-up was calculated from HCT until death from any cause or last follow-up (censoring of survivors).

Above computations relied on standard software (SPSS Statistics v25 IBM, Chicago, IL, USA; Stata SE v16 StataCorp LLC, College Station, TX, USA). All *p* values are two-sided and *p* < 0.05 was considered statistically significant.

## Results

### Patient characteristics

During the study period, 632 patients underwent an allo-HCT. Exclusion criteria are patients less than 18 years old (*n* = 30), GvHD prophylaxis with posttransplant cyclophosphamide on day +3 and +4 (*n* = 31 haploidentical donors and *n* = 23 mismatched unrelated donors) and therefore CsA start on day +5 after allo-HCT or no CsA application (*n* = 13). Moreover, patients with bone marrow failure (n = 13) and cord blood graft source (*n* = 3) were excluded from the analyses. Of these, 113 patients were excluded because of mentioned exclusion criteria and 519 patients were included in the final analysis. The patient- and transplant-related characteristics are described in Table 1: The median age of the entire patient cohort was 54 years (IQR, 43–63 years). The most common underlying hematological disease was myeloid neoplasm (67%), followed by lymphoid malignancies (33%). Three hundred sixty-six (71%) patients received a CsA starting dose of 3 mg/kg/d and 153 (29%) patients a starting dose of 5 mg/kg/d. Patients in the CsA3 group compared to the CsA5 group were significantly younger with a median age of 53 years (IQR, 43–62 years) vs. median of 57 years (IQR, 44–65 years), respectively. Main hematological diseases were myeloid malignancies and significantly higher in the CsA5 groups (80% vs. 62%, respectively; *p* < 0.001). The DRI with intermediate to very high within CsA3 vs. CsA5 group was 82% vs. 75%, respectively (*p* = 0.002). Mostly frequently used conditioning regimens were myeloablative with 75% in the CsA3 compared to 60% in the CsA5 group (*p* < 0.001). In line, TBI was more frequently applied in the CsA3 group compared to the CsA5 group (*p* = 0.002). Whereas no differences were seen according donor type in both CsA starting dose groups. In line, frequencies of allo-HCT from 10/10 HLA-identical donor were comparable in both CsA groups (82% in the CsA3 group vs. 85% in the CsA5 group). Stem cell source was mainly peripheral blood and balanced in both CsA starting doses. At baseline, the median eGFR levels according to the CKD-EPI formula in the CsA3 vs. CsA5 group was 105 ml/min/1.73m^2^ (IQR, 92–117 ml/min/1.73m^2^) vs. 101 ml/min/1.73m^2^ (IQR, 84–114 ml/min/1.73m^2^, *p* value = 0.059), respectively (Supplementary Table S1). Accordingly, patients with CKD stages 1 vs. 2 vs. 3 were evident in 79% vs. 16% vs. 5%, respectively in the CsA3 groups compared to 70% vs. 24% and 6%, respectively, in the CsA5 group. There was no initial need to perform starting dose adjustments (only in case of renal toxicity during the course of post-transplantation period). In line, in patients with higher CsA starting doses no clinical relevant decrease in renal function after seven days post HCT could be observed (Supplementary Table S1).

**Table 1:** Patient- and transplant-related characteristics of all patients (*n* = 519) and different cyclosporine therapy groups with 3 mg/kg/d vs. 5 mg/kg/d.

Variable	All patients (*n* = 519), n (%)	3 mg group (*n* = 366), n (%)	5 mg group (*n* = 153), *n* (%)	*p* values
Age (years, median (IQR))	54 (43–63)	53 (43–62)	57 (44–65)	0.024
Diagnosis				<0.001
Myeloid malignancy	350 (67)	228 (62)	122 (80)	
Lymphoid malignancy	169 (33)	138 (38)	31 (20)	
Disease risk index				0.002
Low	105 (20)	67 (18)	38 (25)	
Intermediate	260 (50)	202 (55)	58 (38)	
High	144 (28)	93 (26)	52 (34)	
Very high	9 (2)	4 (1)	5 (3)	
Conditioning regimen				<0.001
Myeloablative	368 (71)	276 (75)	92 (60)	
Reduced intensity	151 (29)	90 (25)	61 (40)	
TBI				0.002
Yes	174 (33)	138 (38)	36 (24)	
No	345 (67)	228 (62)	117 (76)	
GvHD Prophylaxis				0.134
CsA	5 (1)	5 (1)	0 (0)	
CsA + MTX	400 (77)	275 (75)	125 (82)	
CsA + MMF	114 (22)	86 (24)	28 (18)	
ATG used	254 (49)	165 (45)	89 (58)	0.007
Sex				0.005
Male	307 (59)	202 (55)	105 (69)	
Female	212 (41)	164 (45)	48 (31)	
Donor type				0.345
Related matched	199 (38)	146 (40)	53 (35)	
Related mismatched	1 (0)	1 (0)	0 (0)	
Unrelated matched	231 (45)	154 (42)	77 (50)	
Unrelated mismatched	88 (17)	65 (18)	23 (15)	
Donor/recipient gender				0.062
Male/male	208 (40)	137 (37)	71 (46)	
Male/female	98 (19)	65 (18)	33 (22)	
Female/female	102 (20)	78 (21)	24 (18)	
Female/male	111 (21)	86 (24)	25 (16)	
Donor/recipient CMV status				0.017
Positive/positive	173 (33)	118 (32)	55 (36)	
Positive/negative	58 (11)	36 (10)	22 (14)	
Negative/positive	116 (22)	95 (26)	21 (14)	
Negative/negative	172 (33)	117 (32)	55 (36)	
HLA identical	430 (83)	300 (82)	130 (85)	0.512
1-Antigen HLA mismatch	87 (17)	64 (18)	23 (15)	
2-Antigen HLA mismatch	2 (0)	2 (0)	0 (0)	
HLA mismatch class				0.483
Class I mismatch (HLA A,B,C)	63 (12)	45 (12)	18 (12)	
Class II mismatch (HLA DRB1, DQB1)	26 (5)	21 (6)	5 (3)	
Graft source				0.203
Bone marrow	35 (7)	28 (8)	7 (5)	
Peripheral blood	484 (93)	338 (92)	146 (95)	
CD34 + cell dose (x10^6^/kg), median (IQR)	7.6 (5.1–9.8)	8.0 (5.6–10.0)	6.39 (4.5–8.0)	<0.001
Interval diagnosis to HCT (months, median, IQR)	9 (5–23)	8 (4–28)	7 (5–19)	0.137
KPS				0.204
90–100%	402 (78)	289 (79)	113 (74)	
<90%	117 (22)	77 (21)	40 (26)	
EBMT Score				0.790
0–1	22 (4)	16 (4)	6 (4)	
2–4	325 (63)	232 (64)	93 (61)	
>4	172 (33)	118 (32)	54 (35)	
HCT-CI				0.853
0–1	312 (60)	220 (60)	92 (60)	
2–4	147 (28)	102 (28)	45 (29)	
>4	60 (12)	44 (12)	16 (11)	

*CMV* Cytomegalovirus, *CsA* Cyclosporine A, *EBMT* European Group for Blood and Marrow Transplantation, *GvHD* Graft-versus-host disease, *HCT* Hematopoietic stem cell transplantation, *HCT-CI* Hematopoietic cell transplantation comorbidity index, *IQR* Interquartile range, *MMF* Mycophenolate mofetil, *MTX* Methotrexate, *KPS* Karnofsky performance status, *HLA* Human leucocyte antigen, *TBI* Total body irradiation.

The median follow-up of all surviving patients was 1153 days (IQR 580–1,861 days). The median follow-up for patients within the CsA5 cohort vs. CsA3 cohort was 565 days (IQR 239–719) vs. 1,661 days (IQR 1,293–2,197 days), respectively.

### Cumulative incidence function and risk factors for acute GvHD and chronic GvHD

**Fig. 1:**
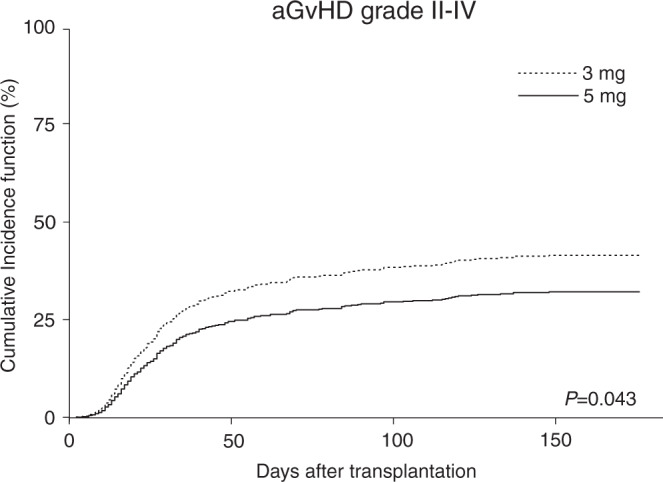
Cumulative incidence function (CIF) of acute graft-versus-host disease (aGvHD) grade II–IV. CIF of aGvHD grade II–IV in patients with 5 mg/kg/d starting dose group (black line) compared to the 3 mg/kg/d starting dose group (dashed line).

The CIF of aGvHD grade ≥2 was higher in patients within the CsA3 compared to the CsA5 group (41% [95% CI: 36–46%] vs. 33% [95% CI: 26–40%], respectively; *p* = 0.043, Table 2, Fig. 1). Additional analyses of CIF of aGvHD grade III–IV in the CsA3 vs. CsA5 cohort were performed: the results revealed that more patients in the CsA3 cohort showed a higher CIF of aGvHD III–IV with 15% (95% CI: 12–19%) compared to patients in the CsA5 (CIF of aGvHD grade III–IV of 10%; 95% CI: 6–15%), respectively. Albeit the analysis of aGvHD grade III–IV failed to reach statistical significance (*p* = 0.082) probably due to the limited statistical power of patients with aGvHD grade III–IV, the analysis also confirm that higher starting of 5 mg/kg/d could also reduce the incidence of aGvHD grade III–IV (Table 2). Following an allo-HCT, there was no significant difference in one-year CIF of cGvHD in patients with 3 mg/kg/d vs. 5 mg/kg/d (41% [95% CI: 36–46%] vs. 38% [95% CI: 30–46%], respectively; *p* = 0.792).

**Table 2:** HCT outcome within different CsA starting doses of 3 mg/kg/d and 5 mg/kg/d.

Variable	3 mg/kg (*n* = 366)	5 mg/kg (*n* = 153)	*p* values
aGvHD II–IV, d180 CIF (95% CI), %	41 (36–46)	33 (26–40)	0.043^a^
aGvHD III–IV, d180 CIF (95% CI), %	15 (12–19)	10 (6–15)	0.082^a^
Acute GvHD steroid refractory, *n* (%)	36 (10)	14 (9)	0.809
Primary graft failure (%)	11 (3)	2 (1)	0.435
1-year CIF of cGvHD (95% CI), %	41 (36–46)	38 (30–46)	0.792^a^
1-year CIF of extensive cGvHD (95% CI), %	29 (24–34)	21 (14–28)	0.126^a^
1-year OS (95% CI), %	70 (65–74)	79 (71–85)	0.034
1-year PFS (95% CI), %	58 (52–63)	66 (58–74)	0.033
1-year NRM CIF (95% CI), %	14 (11–18)	8 (5–14)	0.075^a^
1-year relapse CIF (95% CI), %	28 (24–33)	25 (18–33)	0.292^a^

*CI* Confidence interval, *CIF* Cumulative incidence function, *aGVHD* Acute graft-versus-host disease, *cGVHD* Chronic graft-versus-host disease; *HCT* Hematopoietic cell transplantation, *d* Day, *NRM* Non-relapse-related mortality, *OS* Overall survival, *PFS* Progression-free survival.

^a^derived univariable competing risk regression.

In a multivariable analysis CsA starting dose of 5 mg/kg/d (subdistribution hazard ratio [SHR]: 0.72, 95% CI: 0.53–0.99; *p* = 0.045), use of ATG (SHR: 0.69, 95% CI: 0.52–0.92; *p* = 0.010) and unrelated donor (SHR: 1.80, 95% CI: 1.33–2.45; *p* < 0.001) were associated with incident aGvHD ≥2 (Table 3).

**Table 3:** Uni- and multivariable competing risk or, where appropriate Cox regression analysis for aGvHD grade ≥2, 1-year -NRM,- relapse and – overall survival for groups with differing CsA starting doses.

	Univariable analysis		Multivariable analysis	
CSA starting dose^a^	SHR/HR (95% CI)	*p* value	SHR/HR (95% CI)	*p* value
aGVHD grade II–IV^b^	0.72 (0.53–0.98)	0.043	0.72 (0.53–0.99)	0.045
1-year NRM^c^	0.57 (0.30–1.06)	0.075	0.58 (0.31–1.09)	0.091
1-year relapse^d^	0.82 (0.56–1.19)	0.296	0.78 (0.53–1.15)	0.206
1-year overall survival^e^	0.64 (0.43–0.97)	0.035	0.63 (0.41–0.95)	0.027

Number of subjects included in the univariable and multivariable model *n* = 519.

*CI* Confidence interval, *CsA* Cyclosporine A, *DRI* Disease risk index, *aGVHD* Acute graft-versus-host disease, *HCT* Hematopoietic cell transplantation, *SHR* Subhazard ratio, *RIC* Reduced intensity conditioning, *NRM* Non-relapse mortality.

^a^3 mg/kg starting dose is baseline HR = 1.

^b^model adjusted for DRI, donor type, ATG use.

^c^model adjusted for DRI, donor type, ATG use.

^d^model adjusted for DRI, donor type, ATG use.

^e^model adjusted for ATG use, DRI, donor type.

### Cyclosporine starting doses and their levels post-allo HCT

The comparison of median CsA levels within different CsA starting doses provide all over higher median CsA levels in the 5 mg/kg/d CsA starting dose compared to 3 mg/kg/d (Supplementary Table S2). In line with our previous data [36], we could confirm that aGvHD grade ≥2 within different CsA cut offs of 50–99 μg/L, 100–149 μg/L, 150-199 μg/L and ≥200 μg/L at day 10 after allo-HCT was at lowest in patients with CsA levels greater or equal than 200 μg/L (*p* < 0.001; data not shown).

### Survival, NRM, and relapse rate

**Fig. 2:**
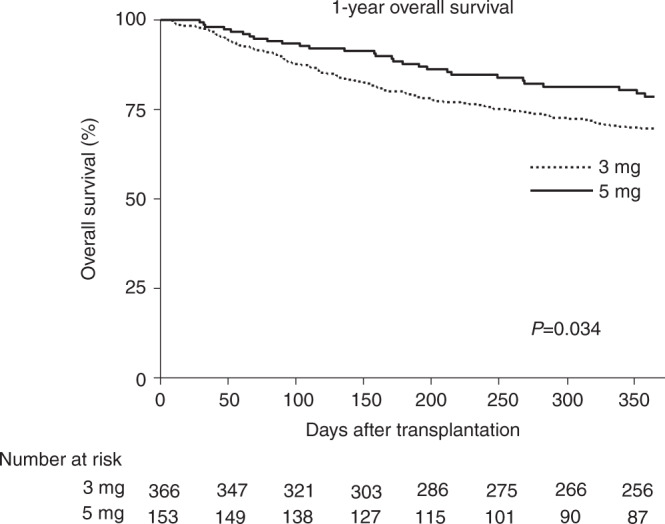
Kaplan–Meier curve for overall survival (OS). One-year OS for CsA 5 mg/kg/d starting dose group (black line) compared to the CsA 3 mg/kg/d starting dose group (dashed line).

As shown in Table 2 and Fig. 2, one-year OS was higher in the 5 mg/kg/d starting dose with 79% (95% CI: 71–85%) compared to patients with CsA 3 mg/kg/d starting dose (70% [95% CI: 65–74%], respectively, *p* = 0.034). As shown, higher overall survival was associated with a non-significantly lower NRM incidence in the 5 mg/kg/d vs. 3 mg/kg/d group with 8% (95% CI: 5–14%) vs. 14 (95% CI: 11–18%; *p* = 0.075), respectively. Moreover, in the multivariable analyses we observed that a higher CsA starting dose and ATG use was associated with higher 1-year OS (adjusted HR 0.63; 95% CI: 0.41–0.95; *p* = 0.027 vs. adjusted HR 0.55; 95% CI: 0.39–0.78; *p* = 0.001), respectively (as depicted in Table 3). CIF of  1-year relapse and -PFS are shown in Table 2 and multivariable analyses for 1-year NRM and -relapse including, CsA starting doses, ATG use, donor type and DRI are shown in Table 3.

## Discussion

The GvHD is a frequent complication after allo-HCT with impact on morbidity and mortality [4, 5]. Continuous and detailed investigations to optimize GvHD prophylaxis are warranted [12, 13]. Calcineurin inhibitors such as CsA have been the backbone of GvHD prophylaxis for decades [20, 21]. Despite long-term experience with CsA, the optimal starting dose to achieve targeted CsA levels in the prevention of GvHD after allo-HCT is still unclear and thus an unmet clinical need [23, 24]. Since previous studies showed that patients with higher CsA levels within the first few weeks post-HCT (ranging between 1 and 4 weeks) had lower incidence of GvHD [22, 26, 28, 36, 48], our study focused on different CsA starting doses of 3 mg/kg/d vs. 5 mg/kg/d on the incidence of GvHD after allo-HCT. There is some credibility to the fact that preventing GvHD is easier than to treat and that possibly early T-cell inhibition may be preferable [49]. Until 2016, the initial starting dose at our center was 3 mg/kg/d targeting trough concentration 150–200 µg/L [50]. In spite of dose adjustments targeted CsA levels were insufficiently reached. Therefore, in 2016, the starting CsA dose was increased to 5 mg/kg/d in order to reach appropriate CsA levels in a greater proportion of patients [36]. As a result, our data indicate that a higher proportion of individuals achieve targeted CsA levels (according previous analyses >195 µg/L in the first 10 days after allo-HCT; [36]) with the higher starting dose of 5 mg/kg/d compared to the starting dose of 3 mg/kg/d [81/153 (53%) vs. 147/363 (40%), respectively; *p* = 0.009, data not shown] and thus a potentially causal link with higher starting dose of CsA with 5 mg/kg/d is likely. Moreover, for every 50 µg/L in level increase on day 10 after allo-HCT a decrease of the CIF of aGvHD was observed, i.e. there is a dose response gradient. Our results are in line with Rogosheske et al. [28] who showed that higher CsA levels above 200 ng/mL, especially in the earlier period within the first two weeks post-transplantation contributed to lower risk of aGvHD.

In comparison to previous studies we found that the incidence of aGvHD was independent of conditioning regimen [17, 51, 52]. Ram et al. found in a large study that higher CsA levels in the first two weeks after allo-HCT lowered the risk of GvHD in patients receiving RIC conditioning regimes, but not in patients treated with MAC [27]. In contrast, Rogosheske et al. demonstrated after adjusting for CsA level, that the risk of aGvHD was similar in patients with different conditioning regimes [28]. Notably, in both studies, the starting dose in RIC was at day -3 with 5 mg/kg/d (i.v.), while in MAC the CsA starting strategy was 3 mg/kg/d at day -1 [27, 28]. In contrast, in our study we start mostly CsA on day -3 in MAC, and on day -1 in RIC if fludarabine/low-dose TBI protocols were used. In line, we could show that the median of CsA levels after allo-HCT was higher in all patients with a starting dose of 5 mg/kg/d, moreover this was also evident in patients treated within MAC protocols (*p* = 0.002, data not shown). The results may indicate that a higher starting dose of 5 mg/kg/d may overcome the potential negative effects of CsA starting dose in patients treated with CsA given at day -3 in MAC. Albeit, previous studies include different conditioning regimes, strategies to start CsA, our policy with higher starting doses on both MAC and RIC regardless of initiation, may explain in parts the independency of target trough CsA levels on aGvHD irrespective of conditioning regimens [27, 28].

Although several side effects of higher CsA levels were described in the literature [25, 53, 54] and dose dependent CsA-related renal toxicity in previous studies are commonly developed, in line with de Kort et al. we found no renal impairment within different CsA starting doses and reached sufficient CsA levels within the first 7 days after allo-HCT [22]. Therefore, our results highlight that close monitoring of CsA levels can avoid renal toxicity also with higher CsA starting doses. Ongoing current investigations will include other side effects of dose depended CsA levels within different intensity of CsA starting doses.

Current studies highlight the promising role of post-transplant cyclophosphamide in combination with cyclosporine as a GvHD prophylaxis (e.g HLA mismatched unrelated donor) in allo-HCT. Whether higher CsA starting doses play a role in the context of post-transplant cyclophosphamide GvHD prophylaxis cannot be determined based on our data [55, 56].

Previous studies showed that higher CsA levels may have an impact on risk of NRM and OS [27, 28]. In contrast to previous studies [22, 26], we found that a higher starting dose with subsequent improved targeted CsA levels may be translated into a higher OS. This may be explained by a lack of power to detect differences or by confounding with variables not measured or observed.

This study has several limitations: Due to the observational nature of our study, treatments could not be allocated randomly. Secondly, we used a heterogeneous patient cohort with different hematological disorders, conditioning and GvHD prophylaxis regimens and duration of follow-up differed among groups. Heterogeneity with small numbers in certain subsets may reduce the generalizability of our results. In spite of careful adjustment in multivariable analysis demonstrating the superiority of the 5 mg/kg starting dose, we cannot exclude unmeasured confounding.

In conclusion, our results indicate that a higher CsA starting dose of 5 mg/kg/d may increase the proportion of patients with desired CsA levels after allo-HCT, which was a preventive factor of incident aGvHD. These results underline the importance of reaching sufficient CsA levels within an early time period following allo-HCT. If these data are confirmed, recommendations as to starting doses currently at 3 mg/kg/d may be amended.

## Supplementary information

Supplementary Information
