# Supplementary material for: Optimized cyclosporine starting dose may reduce risk of acute GvHD after allogeneic hematopoietic cell transplantation: a single-center cohort study
Source: Bone Marrow Transplant. 2022 Feb 8;57(4):613–9. doi: 10.1038/s41409-022-01598-6 (PMC8993684; doi:10.1038/s41409-022-01598-6)
Supplement: Supplementary file 1 — Supplementary Information [file 41409_2022_1598_MOESM1_ESM.pdf]

Supplementary Table S1. Baseline and follow up of renal function within different CsA starting doses

Variable	3mg/kg (n=366)	5mg/kg (n=153)	p-value
**CKD-staging at baseline, CKD Stages, n (%)**			0.097
Stage 1 (eGFR ≥90)	288 (79)	107 (70)	
Stage 2 (eGFR 60-89)	59 (16)	36 (24)	
Stage 3 (eGFR 30-59)	19 (5)	10 (6)	
Stage 4 (eGFR 15-29)	0 (0)	0 (0)	
Stage 5 (eGFR ≤15)	0 (0)	0 (0)	
**CKD-staging d 7 post-HCT, CKD-Stages, n (%)**			0.104
Stage 1 (eGFR ≥90)	270 (74)	99 (65)	
Stage 2 (eGFR 60-89)	70 (19)	45 (29)	
Stage 3 (eGFR 30-59)	23 (6)	9 (6)	
Stage 4 (eGFR 15-29)	2 (1)	0 (0)	
Stage 5 (eGFR ≤15)	1 (0)	0 (0)	
**Serum creatinine at baseline (µmol/l), median (IQR)**	59 (50-75)	64 (52-81)	0.022
**eGFR (CDK-EPI, ml/min/1.73m^2), median (IQR)**			
Day 0	105 (92-117)	101 (84-114)	0.059
Day 7	102 (88-114)	98 (83-112)	0.054

Abbreviation: CDK: chronic kidney disease, CsA: Cyclosporine A, eGFR: estimated glomerular filtration rate, HCT: hematopoietic stem cell transplantation, KDIGO: kidney disease: improving global outcome

Supplementary Table S2: Median CsA levels in different CsA starting doses of 3mg/kg/d and 5mg/kg/d at day0 until day30 after HCT

	3mg/kg/d	5mg/kg/d	No. of Individuals with missing data
**Day**	**CsA median (IQR)**	**CsA median (IQR)**	
**d0**	114 (85-153)	128 (100-169)	42
**d2-4**	140 (107-182)	162 (128-204)	64
**d10**	179 (147-218)	200 (161-242)	3
**d17**	214 (173-270)	234 (195-294)	25
**d24**	203 (160-256)	219 (176-295)	36
**d30**	182 (133-239)	188 (134-248)	47
